# Tumor-Derived Sarcopenia Factors Are Diverse in Different Tumor Types: A Pan-Cancer Analysis

**DOI:** 10.3390/biomedicines12020329

**Published:** 2024-01-31

**Authors:** Xin Gan, Yunqian Zeng, Jiaquan Huang, Xin Chen, Hao Kang, Shuaiwen Huang

**Affiliations:** 1 Department of Orthopedics, Tongji Hospital, Tongji Medical College, Huazhong University of Science and Technology, Wuhan 430030, China; xingan@hust.edu.cn (X.G.); m202176390@hust.edu.cn (Y.Z.); m202276376@hust.edu.cn (X.C.); 2 Department and Institute of Infectious Disease, Tongji Hospital, Tongji Medical College, Huazhong University of Science and Technology, Wuhan 430030, China; huangjiaquan21@aliyun.com; 3 Department of General Practice, Tongji Hospital, Tongji Medical College, Huazhong University of Science and Technology, Wuhan 430030, China

**Keywords:** tumors secreting, cancer-associated muscle wasting, The Cancer Imaging Archive, pan-cancer analysis, TCGA, tumor-derived factors

## Abstract

Cancer-associated muscle wasting is a widespread syndrome in people with cancer and is characterized by weight loss and muscle atrophy, leading to increased morbidity and mortality. However, the tumor-derived factors that affect the development of muscle wasting and the mechanism by which they act remain unknown. To address this knowledge gap, we aimed to delineate differences in tumor molecular characteristics (especially secretion characteristics) between patients with and without sarcopenia across 10 tumor types from The Cancer Genome Atlas (TCGA). We integrated radiological characteristics from CT scans of TCGA cancer patients, which allowed us to calculate skeletal muscle area (SMA) to confirm sarcopenia. We combined TCGA and GTEx (The Genotype-Tissue Expression) data to analyze upregulated secretory genes in 10 tumor types compared with normal tissues. Upregulated secretory genes in the tumor microenvironment and their relation to SMA were analyzed to identify potential muscle wasting biomarkers (560 samples). Meanwhile, their predictive values for patient survival was validated in 3530 samples in 10 tumor types. A total of 560 participants with transcriptomic data and SMA were included. Among those, 136 participants (24.28%) were defined as having sarcopenia based on SMA. Enrichment analysis for upregulated secretory genes in cancers revealed that pathways associated with muscle wasting were strongly enriched in tumor types with a higher prevalence of sarcopenia. A series of SMA-associated secretory protein-coding genes were identified in cancers, which showed distinct gene expression profiles according to tumor type, and could be used to predict prognosis in cancers (*p* value ≤ 0.002). Unfortunately, those genes were different and rarely overlapped across tumor types. Tumor secretome characteristics were closely related to sarcopenia. Highly expressed secretory mediators in the tumor microenvironment were associated with SMA and could affect the overall survival of cancer patients, which may provide a valuable starting point for the further understanding of the molecular basis of muscle wasting in cancers. More importantly, tumor-derived pro-sarcopenic factors differ across tumor types and genders, which implies that mechanisms of cancer-associated muscle wasting are complex and diverse across tumors, and may require individualized treatment approaches.

## 1. Introduction

Cancer-associated muscle wasting is a widespread syndrome in people with cancer, and is characterized by weight loss and muscle atrophy, leading to increased morbidity and mortality [1]. Patients with muscle wasting often display weakness and frailty, causing loss of independence, a reduced tolerance to treatments, including surgery and chemotherapy, an impaired immune function, and an increased susceptibility to infection [2,3,4,5].

Cancer-associated muscle wasting is a complex pathological condition involving numerous organs. However, a large number of early studies on cancer-associated muscle wasting focused on circulating inflammation mediators and muscle tissue [6,7].

Insufficient attention has been paid to the role of the tumor itself in muscle wasting [7,8,9,10,11]. Recently, several studies have investigated the effect of tumor secretion on tissue metabolic changes [12,13]. Secreted proteins are proteins that are synthesized inside the cell and secreted outside the cell to function. There are growing shreds of evidence that tumors may serve as the source of the mediators that cause muscle mass loss, and muscle wasting may be initiated and/or maintained by secretions from the tumor cells.

However, which tumor-derived factors affect the development of cancer-associated muscle wasting and the mechanism by which they act are still unknown. Clarifying the tumor-derived factors that induce muscle wasting can be helpful for the further understanding of the pathogenesis and developing new targeted treatments/biomarkers of cancer-associated muscle wasting.

While some reports have highlighted the link between muscle wasting and tumor biology in specific tumor types, there is still a dearth of pan-cancer studies. To address this knowledge gap, we aimed to delineate differences in tumor molecular characteristics between patients with and without sarcopenia across 10 tumor types from The Cancer Genome Atlas (TCGA). Particularly, we emphasized the tumor secretion characteristics and explored their predictive values for patient survival. If muscular atrophy of different tumor types may be mediated by the same tumor-derived mediators, it will lay the foundation for the development of broad-spectrum therapeutic agents for cancer-associated muscle wasting. We hypothesized and expected that tumor-driven or secreted mediators associated with sarcopenia would cross and overlap between different tumor types.

## 2. Materials and Methods

### 2.1. Data Sources and Study Population

Data were obtained from TCIA (The Cancer Imaging Archive) and TCGA (The Cancer Genome Atlas). The National Institute of Health/National Cancer Institute’s approved TCGA/TCIA databases contain publicly available datasets in which all data are anonymized. Following the 1964 Declaration of Helsinki and its subsequent revisions or similar ethical standards, institutional review boards of all participating institutions approved informed consent from patients and collected publicly available data.

**Figure 1:**
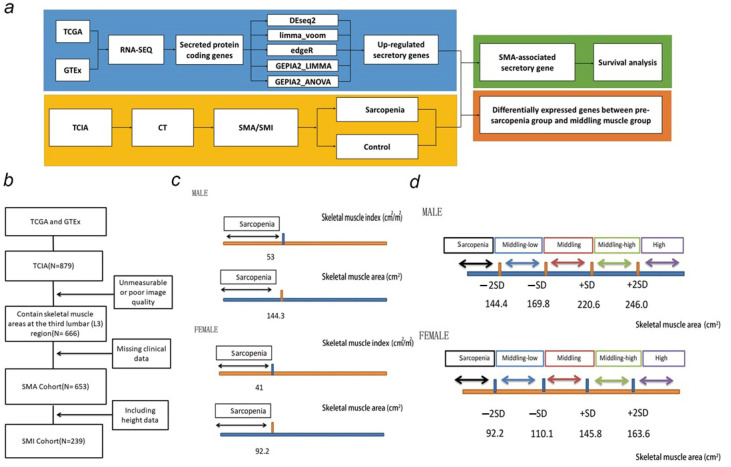
Definitions of five muscle mass patterns and sarcopenia. (**a**) Workflow chart of key steps in this study. (**b**) Flow chart of inclusion and exclusion of study participants. (**c**) Definitions of sarcopenia by SMA (skeletal muscle area)/SMI (skeletal muscle mass index). (**d**) Definitions of five muscle mass patterns by SMA.

The workflow chart of key steps in this study are shown in Figure 1a. In the SMA cohort analysis, subjects with poor image quality or unmeasurable images were excluded, and subjects missing clinical data or transcriptomic data were excluded (Figure 1b). Therefore, a total of 560 participants remained (with transcriptomic and SMA data) for analysis.

### 2.2. Assessment of Muscle Cross-Sectional Area from CT

The CT image data were downloaded from the TCIA website. The baseline CT image before surgery or biopsy was taken, and the cross-sectional area of the mid-third lumbar was calculated using Image J software (V.1.52). SMA was measured as the area of pixels between −29 and +150 Hounsfield Units (HU) in the region of interest on the axial slice, as previously validated [14,15,16]. Muscle cross-sectional areas (SMAs) at the mid-third lumbar for 10 types of tumors were calculated under the guidance of two skilled radiologists. Clinical data and covariates were obtained from the TCGA data portal (https://tcga-data.nci.nih.gov/tcga/, accessed on 1 June 2021) and TCGA-Clinical Data Resource [17].

Using data from a healthy US population [18], we defined five patterns for subjects: the sarcopenia group, the middle–low muscle mass group, the middle muscle mass group, the middle–high muscle mass group, and the high muscle mass group (Figure 1c). Based on EWGSOP, sarcopenia was characterized by low muscle mass, divided by SMA less than 2 SD below the sex-specific mean for young adults [19]. The cut-off value of SMI (skeletal muscle mass index) for sarcopenia was divided by 53 cm/m in males and 41 cm/m in females as described in previous studies (Figure 1d) [20].

### 2.3. Transcriptome-Based Secretome Analysis

Transcriptome data were downloaded from UCSC XENA (http://xena.ucsc.edu/, accessed on 1 June 2021), and transcriptional profiles from TCGA of 10 human cancers were compared with matched normal tissues from TCGA and GTEx. Differentially expressed genes were further filtered for secreted protein-coding genes based on the human secretome list available at The Human Protein Atlas [21]. DEseq2, limma_voom, and edgeR were used for selecting the differentially expressed secreted protein-coding genes. We set the threshold fold change as 1.0 and defined the adjusted *p*-value cutoff as 0.05. To ensure the robustness of the results, GEPIA2 web tools were also used to identify the differentially expressed genes (DEGs) [22]. The VENN tool (http://bioinformatics.psb.ugent.be/webtools/Venn/, accessed on 1 June 2021) was used to identify the overlapped differentially expressed secreted protein-coding genes. Circos (http://circos.ca/, accessed on 1 June 2021) was used to show the upregulated secretory genes shared across tumor types [23]. The KEGG (Kyoto Encyclopedia of Genes and Genomes) and GO (Gene Ontology) pathway of upregulated secretory genes was analyzed by enrichR links (http://amp.pharm.mssm.edu/Enrichr/, accessed on 1 June 2021) [24]. The survival map was drawn using GEPIA2 to show the survival analysis result based on multiple cancer types [22]. Risk assessment and survival analyses were performed on 9 TCGA tumor datasets using SurvExpress [25].

### 2.4. Statistical Analysis 

Student’s *t*-test and analysis of variance (ANOVA) test were used for comparisons between 2 groups and comparisons among >2 groups, respectively. Pearson or Spearman correlation analyses were used to gauge the degree of correlation between certain variables. The multivariate Cox proportional hazards model was used to assess independent risk factors for survival. The possible non-linear relationships were investigated by nonparametric restricted cubic splines, and *p* < 0.05 was considered statistically significant. All statistical tests are performed using IBM SPSS software (Version 22, Armonk, NY, USA) and R software (version 4.0.3; R Foundation for Statistical Computing, Vienna, Austria).

## 3. Results

### 3.1. Baseline and SMA Characteristics of TCGA Samples

**Figure 2:**
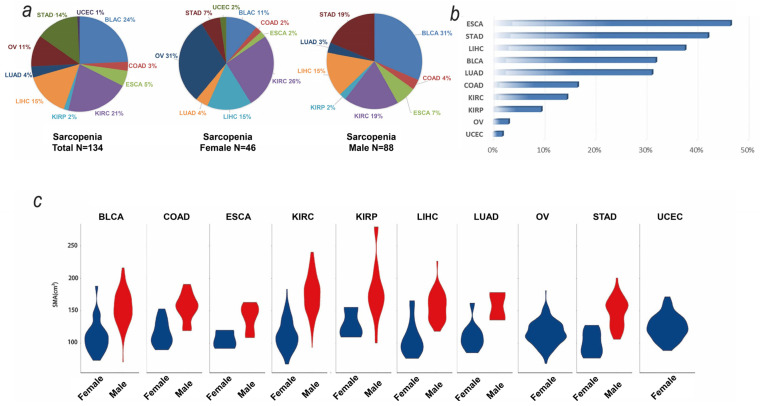
Distribution of SMA and its relationship with prognosis. (**a**) Distribution of sarcopenia in tumors. (**b**) Boxplot diagram of the SMA of each tumor. (**c**) Violin diagram of the SMA of each tumor. Abbreviations: BLAC: bladder cancer; COAD: colon cancer; ESCA: esophageal cancer; KIRC: kidney clear cell carcinoma; KIRP: kidney papillary cell carcinoma; LIHC: liver cancer; LUAD: lung adenocarcinoma; OV: ovarian cancer; STAD: stomach cancer; UCEC: endometrioid cancer.

A total of 560 participants with transcriptomic data and muscle cross-sectional area (SMA) at the mid-third lumbar were included (Table 1). The general information of the included tumor participants is shown in Table S1 and the calculated SMA/SMI data for 10 types of tumors are shown in Tables S2–S4. Among these, 136 participants were defined as displaying sarcopenia based on their SMA. The distribution of sarcopenia based on SMA in tumors is shown in Figure 2a. According to the definition of sarcopenia, we compared the prevalence of sarcopenia among different tumors. ESCA (esophageal carcinoma), LIHC (liver hepatocellular carcinoma), and STAD (stomach adenocarcinoma) showed the highest prevalence of sarcopenia (Figure 2b). The violin diagram of the SMA of each tumor is shown in Figure 2c.

Next, we aimed to analyze the association between SMA and prognosis. As shown in Figure S1a, there was a significant correlation between muscle cross-sectional area (SMA) and prognosis in tumor patients. Sarcopenia was significantly associated with increased risk of mortality after adjusting for age, sex, stage, and race (Figure S1b). The non-linear associations between SMA and mortality were further confirmed by the restricted cubic spline (Figure S2). 

### 3.2. Upregulated Secreted Protein-Coding Genes and Related Pathways in Cancers

**Figure 3:**
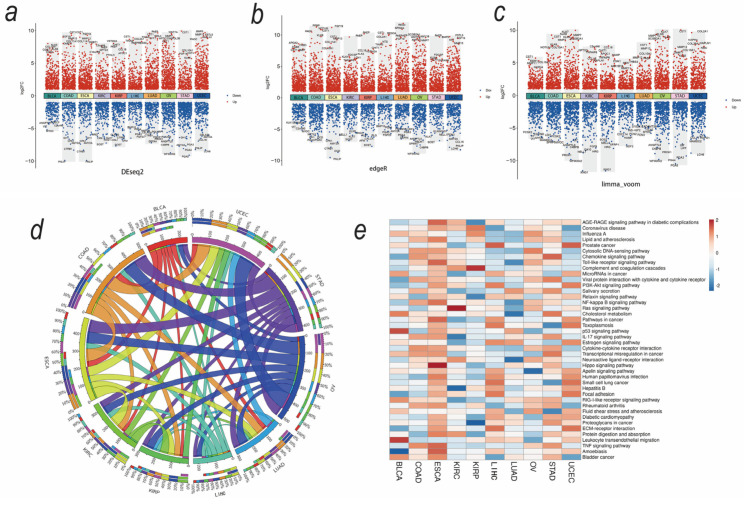
Identification of differentially expressed secretome genes and related gene pathways. (**a**–**c**) Differentially expressed secretome genes across cancers generated by DEseq2, limma_voom, and edgeR. (**d**) The thickness of each link in the Circos plot represents the number of shared upregulated secretome genes among tumor types. (**e**) Differentially expressed secretome genes related to KEGG pathways are shared among tumor types.

Given that many secreted soluble factors are associated with muscle wasting in different tumor types, significant upregulation of secreted protein-coding genes may reveal key factors in the pathogenesis of the syndrome. Gene expression profiles were obtained from 3530 tumors comprising 10 cancer types (TCGA) and 1720 corresponding normal tissues (TCGA and GTEx). A summary of the number of TCGA and GTEx samples is described in Table S5. The secretome genes showed differential expression profiles across cancers (Figure 3a–c). The Venn map shows the differential secretory protein-coding genes identified by different methods (Figure S3). The upregulated secretory protein-coding genes (using five methods) are listed in Table S6. 

In Figure 3d, the thickness of each link in the Circos plot represents the number of upregulated genes shared across tumor types. Tumors with a higher prevalence of sarcopenia shared more upregulated secreted protein-coding genes. Using the KEGG pathways and Gene Ontology terms, we performed a functional enrichment analysis of those upregulated genes. Those pathways associated with muscle wasting, such as extracellular matrix–receptor interaction, protein digestion and absorption; PI3K-Akt signaling, TNF signaling; and NK-kappa B signaling, were strongly enriched in tumors with a higher prevalence of sarcopenia (ESCA, LIHC, and STAD) (Figure 3e). These results suggest that tumor secretome characteristics are closely related to sarcopenia.

### 3.3. SMA-Associated Upregulated Secretory Protein-Coding Genes and Their Predictive Values for Patient Survival

**Figure 4:**
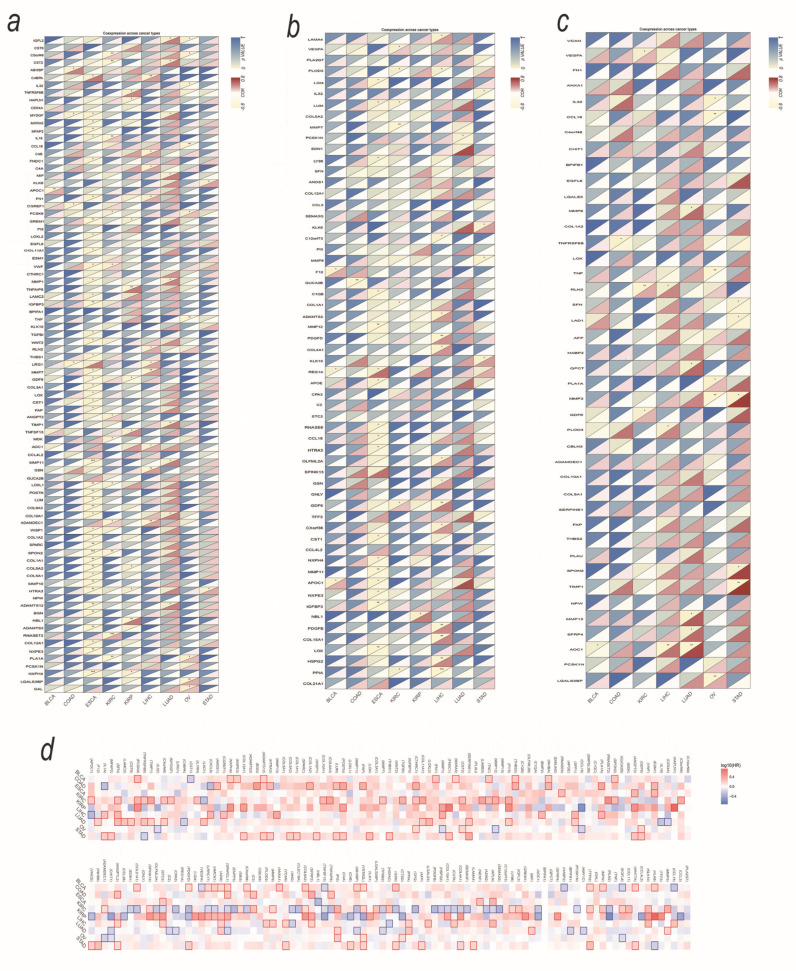
Relations of SMA with expression of the secretory protein-coding genes and the predictive values of SMA-associated secretory protein-coding genes. (**a**) The pan-cancer correlations between SMA and expression of the secretory protein-coding genes in the SMA cohort. (**b**) The pan-cancer correlations between SMA and expression of the secretory protein-coding genes in males. (**c**) The pan-cancer correlations between SMA and expression of the secretory protein-coding genes in females. (**d**) Gene survival map of SMA-associated secretory protein-coding genes. * *p* value < 0.05; ** *p* value < 0.01; *** *p* value < 0.001.

Next, we analyzed the association between upregulated genes with SMA, trying to clarify if there is a universal panel of the secretory protein-coding genes of sarcopenia caused by different tumor types, which would lay the foundation for the development of possible broad-spectrum therapeutic agents. Therefore, we calculated the Spearman and Pearson correlation coefficients between upregulated secretory protein-coding gene expression and SMA in each tumor (Table S7). The pan-cancer correlations between SMA and expression of the upregulated secretory protein-coding genes are displayed in Figure 4a, which show that the upregulated secretory protein-coding genes were closely associated with SMA. 

Considering the gender differences of SMA, Figure 4b,c show the correlations in males and females, respectively. The results show that male patients have many more genes associated with SMA, implicating that male patients may exhibit a higher risk of sarcopenia. Genes significantly associated with SMA were further included in the survival analysis (data from TCGA including 3530 samples). The gene survival map demonstrates the effect of these genes on tumor prognosis (Figure 4d).

**Figure 5:**
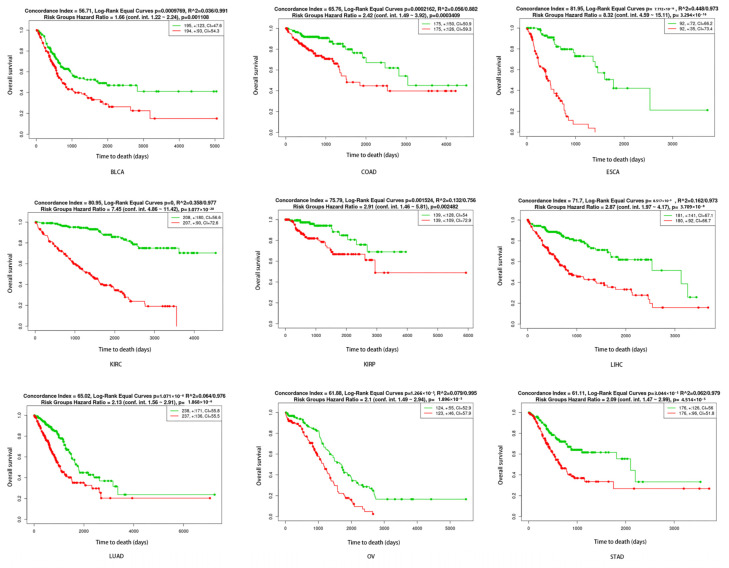
The expression landscape of SMA-associated secretory protein-coding genes predicts cancer outcome. Survival analysis based on the tumor mRNA expression of SMA-associated secretory protein-coding genes. The data were calculated using the datasets from The Cancer Genome Atlas of 9 tumor types in the web-based tool SurvExpress, which stratified the cancer patients into high-risk or low-risk groups (red and green, respectively).

Risk and prognosis models were constructed using SMA-associated upregulated secretory protein-coding genes (Table S8) by SurvExpress. SurvExpress generated a prognostic index (risk score) based on the SMA-associated upregulated secretory protein-coding genes and the survival of cancer patients for each of the nine tumor types (UCEC was removed due to too small of a sample size). Patients were divided into two groups, high risk and low risk, maximizing the number of patients into risk groups by an optimization algorithm from the ordered prognostic index. The distinct SMA-associated gene expression profile, according to tumor type, could be used to predict prognosis in cancers (*p* value ≤ 0.002), as shown in Figure 5. 

### 3.4. Tumor-Derived Sarcopenia Factors Are Diverse in Different Tumor Types and Genders

**Figure 6:**
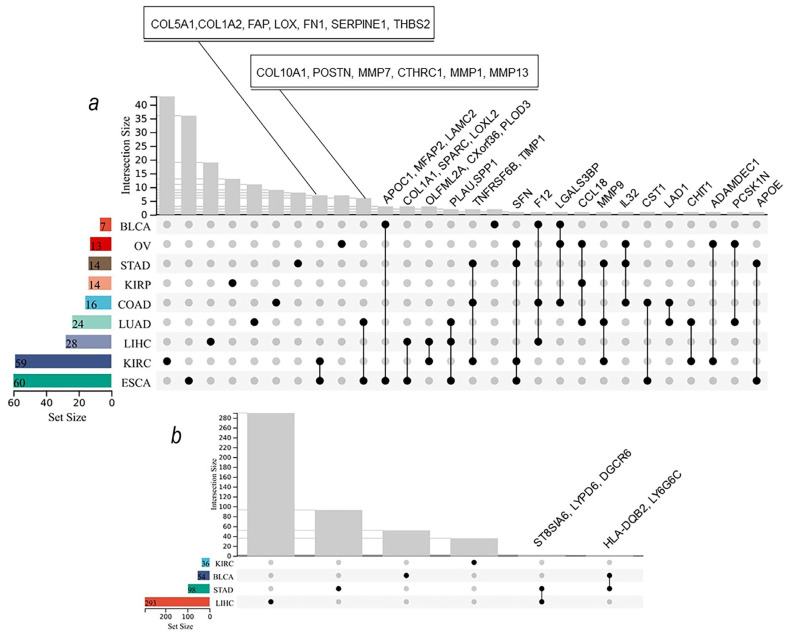
Upregulated secretory protein-coding genes associated with SMA (**a**) and differentially expressed genes in the sarcopenia and middling muscle mass groups (**b**) were different and rarely overlapped across tumor types. Grey dots indicate that they are not included in the corresponding dataset, while black dots indicate that they are included in the corresponding dataset.

We further compared the upregulated secretory protein-coding genes across tumor types to find a universal panel to identify patients with sarcopenia regardless of tumor types. Unexpectedly, those upregulated secretory protein-coding genes associated with SMA were different and rarely overlapped across tumor types (Figure 6a). This indicates that tumor-derived sarcopenia factors were diverse in different tumor types, and therefore, the treatment of sarcopenia caused by different tumor types should be conducted differently. Meanwhile, more research is needed to reveal the mask of the underlying mechanism. Differentially expressed genes (Table S9) in the sarcopenia and middling muscle mass groups were identified by the DEseq2, limma_voom, and edgeR methods in LIHC (liver hepatocellular carcinoma), STAD (stomach adenocarcinoma), KIRC (kidney renal clear cell carcinoma), and BLCA (bladder urothelial carcinoma) (tumors were selected with consideration of sample size). The differentially expressed genes of these four tumors rarely overlapped, which further confirmed that tumor-derived sarcopenia factors were diverse in different tumor types (Figure 6b). This suggests that the tumor types must be taken into account during the development and clinical application of drugs for sarcopenia. Further, we performed a functional enrichment analysis of these DEGs using the KEGG pathways and Gene Ontology terms, which showed that the enriched pathways were different in different tumor types, including intracellular signaling, immune response, and metabolism (Figure S4).

## 4. Discussion

Many studies have focused on the effect of muscle wasting on tumor prognosis and treatment responses, but the molecular basis has remained unclear [8,9,10,11]. There is growing evidence that tumors are the source of the mediators that cause muscle mass loss, and muscle wasting may be initiated and/or maintained by secretions from the tumor cells [12,13]. But little is known about the common mechanism of muscle wasting across different cancers; therefore, pan-cancer studies are needed to provide key evidence to clarify the association between secretory mediators and the development of sarcopenia. By applying a comprehensive pan-cancer analysis, we aimed to find muscle wasting-related secreted protein-coding genes across different cancer types. Firstly, we quantified the skeletal muscle area (SMA) at the lumbar level mid-L3 of tumor patients using CT images provided in TCIA, making it possible to uncover tumor-derived factors related to muscle wasting. Additionally, we analyzed upregulated secretory protein-coding genes in different tumor types, finding that a variety of secretory mediators highly expressed in the tumor microenvironment were enriched in the pathways closely associated with muscle wasting reported previously, including extracellular matrix–receptor interaction, protein digestion and absorption, PI3K-Akt signaling; TNF signaling; and NK-kappa B signaling. These results were partially in agreement that secretory mediators might play a key role in the development of sarcopenia across cancer types. Understanding and targeting the secretome of cachectic patients will likely represent a promising strategy to preserve muscle during cancer cachexia, thereby enhancing recovery.

To further identify potential muscle wasting biomarkers, we identified a variety of upregulated secretory mediators associated with SMA and found that these genes could affect the overall survival of patients in some types of cancer. This result indicated that secretory mediators might serve as biomarkers and treatment targets for muscle wasting in certain cancers. Risk and prognosis models were constructed using SMA-associated upregulated secretory protein-coding genes, and the distinct SMA-associated gene expression profiles, according to tumor type, could be used to predict prognosis in cancers. Taken together, all of these results provided key evidence for the role of secretory mediators in muscle wasting of cancers, and targeting certain genes in separate cancer types might decrease the incidence of sarcopenia. 

Further, we compared the SMA-associated upregulated secretory protein-coding genes across tumor types. Unexpectedly, those SMA-associated upregulated secretory protein-coding genes were different and rarely overlapped across tumor types, indicating that tumor-derived sarcopenia factors were diverse in different tumor types. Also, gender played a role in the association between secretory protein-coding genes, where male patients exhibited more associated genes in pan-cancers. This could partially explain the reason that male patients with cancer have a poor prognosis compared with females. All of these data implied that mechanisms of cancer-associated muscle wasting are complex and diverse across tumors and gender, and therefore individualized treatment approaches are needed in sarcopenia. Also, the development and clinical application of drugs for sarcopenia may need to take these two factors into account.

We found a few genes that overlapped between two or three types of cancer, which may serve as valuable targets for broad-spectrum therapeutic agents in future studies. For example, LAD1, a gene co-located with actin filaments, filamentous proteins, and actin cross-linking cytoskeletal proteins [26], was found to be associated with SMA and could affect the overall survival of both KIRC and LUAD. This could partially explain previously published observations that showed the relationship between LAD1 and tumor prognosis [27,28]. In addition, we found that TIMP1 was associated with SMA and could affect the overall survival both in COAD and LUAD. This was in line with existing data, which showed that plasma TIMP1 levels were positively associated with clinical markers of cachexia (including absolute and relative values of weight loss and lung function, as well as ferritin, hemoglobin, and cholinesterase levels) in patients with chronic pancreatitis and pancreatic cancer [29]. Still, the association between the expression of these genes and sarcopenia needs to be confirmed by further studies.

To our knowledge, our study measured the muscle cross-sectional area at the level of the mid-third lumbar spine in TCGA patients and innovatively matched it with transcriptome data in a pan-cancer analysis. This provides a valuable starting point for further understanding of the role of the tumor itself in cancer-associated muscle wasting. Further, our study enhances our understanding of the relationship between SMA-associated upregulated secretory protein-coding genes with the outcome of cancer patients. These results lay a foundation for further analysis of the mechanism of muscle wasting and its early detection. In addition, we jointly analyzed the TCGA and TCIA data and shared the measured SMA/SMI data (Tables S2–S4), which provided data to support further understanding of the mechanism of tumor-related muscle wasting. It also provides convenience for subsequent researchers.

Several limitations existed in this study, including the relatively small sample size and the unadjusted confounders (such as muscle function) affecting muscle wasting owing to a lack of data. Clinical validation is also needed in future studies.

## 5. Conclusions

In conclusion, we identified a series of SMA-associated secretory protein-coding genes which could be used to predict the prognosis of tumors and provide corresponding biomarkers for the early detection of tumor-associated muscle wasting. Our results provided a valuable starting point for the further understanding of the relationship between secretory proteins and muscle wasting in cancers. More importantly, tumor-derived sarcopenic factors differ across tumor types and genders, which implies that mechanisms of cancer-associated muscle wasting are complex and diverse, and may require individualized treatment approaches.

## Figures and Tables

**Table 1:** Data obtained from TCIA and TCGA.

Cancer Type ^$^	Total ^#^	SMA Data *	Clinical Data ^&^	Transcriptomic Data ^!^
TCGA-UCEC	65	52	52	3
TCGA-STAD	46	45	45	43
TCGA-OV	143	129	128	96
TCGA-LUAD	67	28	16	16
TCGA-LIHC	97	53	53	53
TCGA-KIRP	33	21	21	21
TCGA-KIRC	267	199	199	196
TCGA-ESCA	16	15	15	14
TCGA-COAD	25	24	24	19
TCGA-BLCA	120	100	100	99
Pan-cancer	879	666	653	560

^$^ UCEC: uterine corpus endometrial carcinoma; STAD: stomach adenocarcinoma; OV: ovarian serous cystadenocarcinoma; LUAD: lung adenocarcinoma; LIHC: liver hepatocellular carcinoma; KIRP: kidney renal papillary cell carcinoma; KIRC: kidney renal clear cell carcinoma; ESCA: esophageal carcinoma; COAD: colon adenocarcinoma; BLCA: bladder urothelial carcinoma. ^#^ Patients with computed tomography data in TCIA data. * Skeletal muscle areas (SMAs) calculated at the third lumbar (L3) region in TCIA data. ^&^ SMA data with clinical date in TCGA-CDR. ^!^ SMA data with transcriptomic data.

## Data Availability

Data supporting reported results can be found in Supplementary Materials. The datasets are also available from the corresponding author upon reasonable request.

## Supplementary Materials

The following supporting information can be downloaded at https://www.mdpi.com/article/10.3390/biomedicines12020329/s1: Figure S1: (a) Kaplan–Meier overall survival curves of patients stratified by the presence of sarcopenia; (b) associations between muscle mass patterns and risk of mortality adjusted for baseline age, race, stage, and sex in SMA cohort. Figure S2: The non-linear associations between SMA and mortality in males (a) and females (b), respectively. Figure S3: Venn map for the upregulated secreted protein-coding genes among tumor types. Figure S4: Differentially expressed genes (DEGs) and related pathways between the sarcopenia group and middling muscle group in LIHC, STAD, KIRC, and BLCA. DEGs are enriched in multiple tumor-related biological pathways involved in intracellular signaling, immune response, and metabolism. Table S1: The general information of SMA cohort. Table S2: Summary SMA/SMI data of TCGA patients. Table S3: Skeletal muscle areas at the mid-third lumbar (L3) region of TCGA patients. Table S4: Skeletal muscle index data of TCGA patients. Table S5: The summary of the number of TCGA and GTEx samples used for determining upregulated secreted protein-coding genes. Table S6: The upregulated secreted protein-coding genes in cancers. Table S7: Correlation coefficients (Spearman and Pearson) between secretory protein-coding gene expression and SMA in cancers. Table S8: Upregulated secretory protein-coding genes associated with SMA. Table S9: Differentially expressed genes in the sarcopenia and middling muscle mass groups.
