# Supplementary figures and images for: Tumor-Derived Sarcopenia Factors Are Diverse in Different Tumor Types: A Pan-Cancer Analysis
Source: Biomedicines. 2024 Jan 31;12(2):329. doi: 10.3390/biomedicines12020329 (PMC10887289; doi:10.3390/biomedicines12020329)

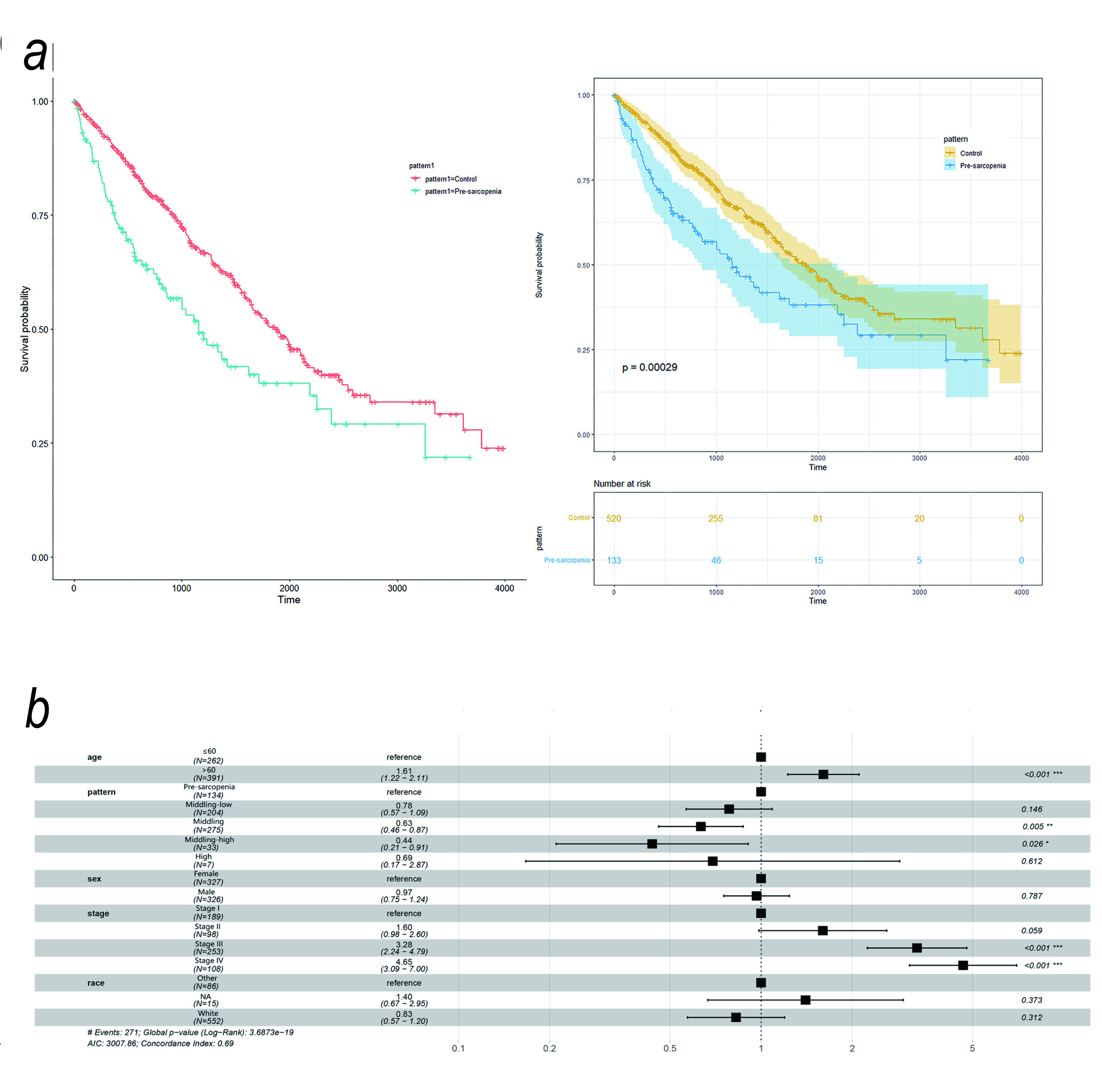

Supplement: Supplementary file 1 [file biomedicines-12-00329-s001.zip › Figure S1.jpg]

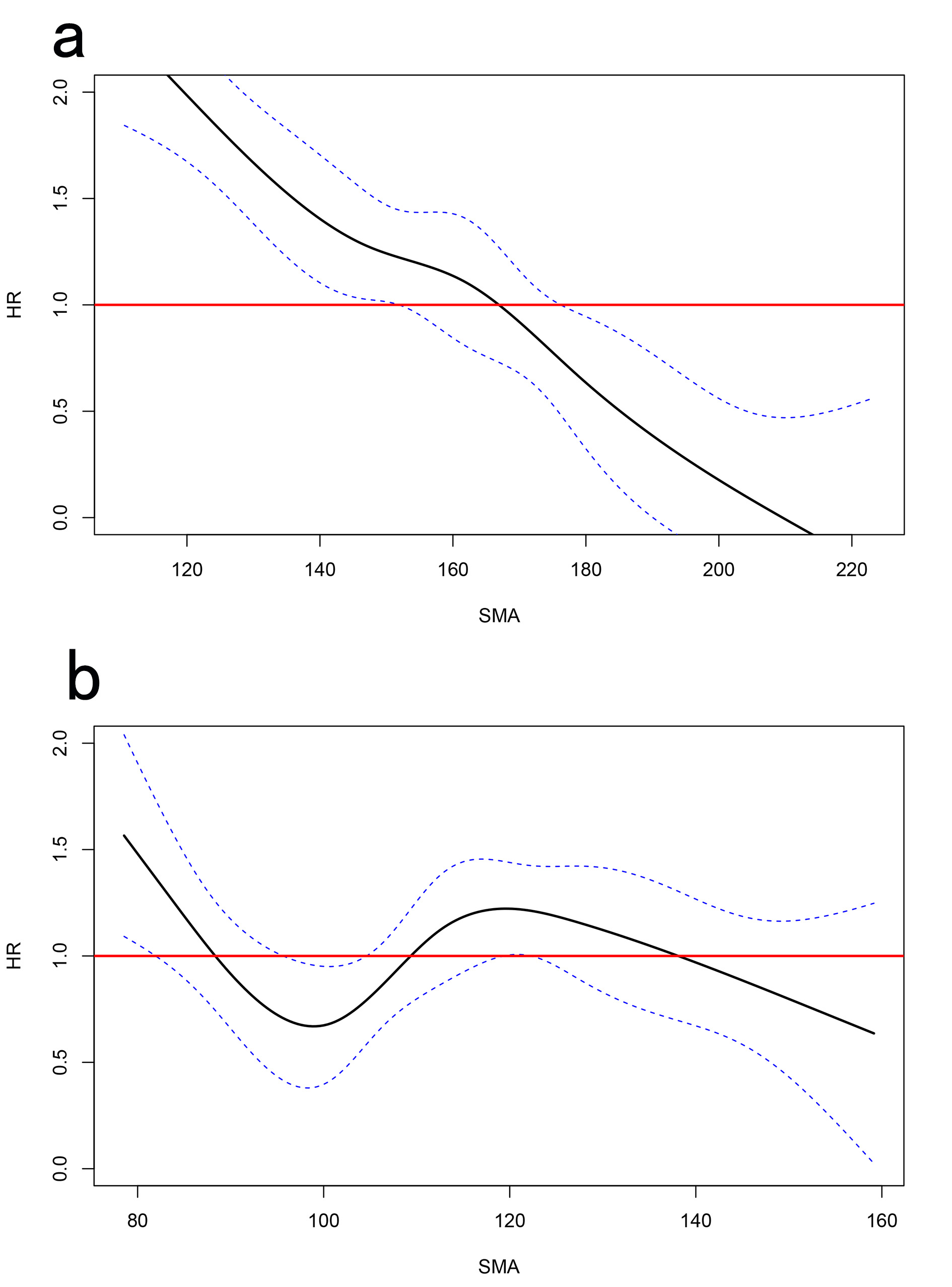

Supplement: Supplementary file 1 [file biomedicines-12-00329-s001.zip › Figure S2.jpg]

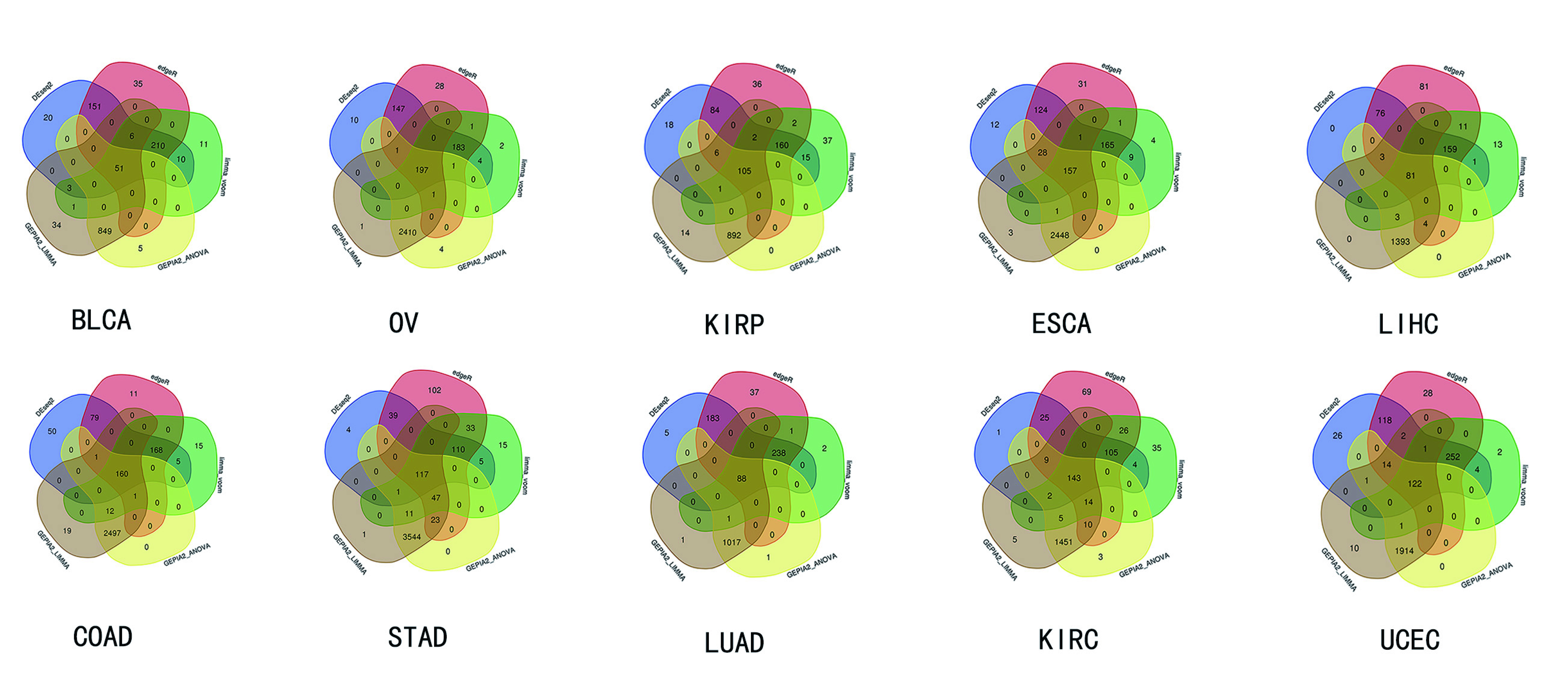

Supplement: Supplementary file 1 [file biomedicines-12-00329-s001.zip › Figure S3.jpg]

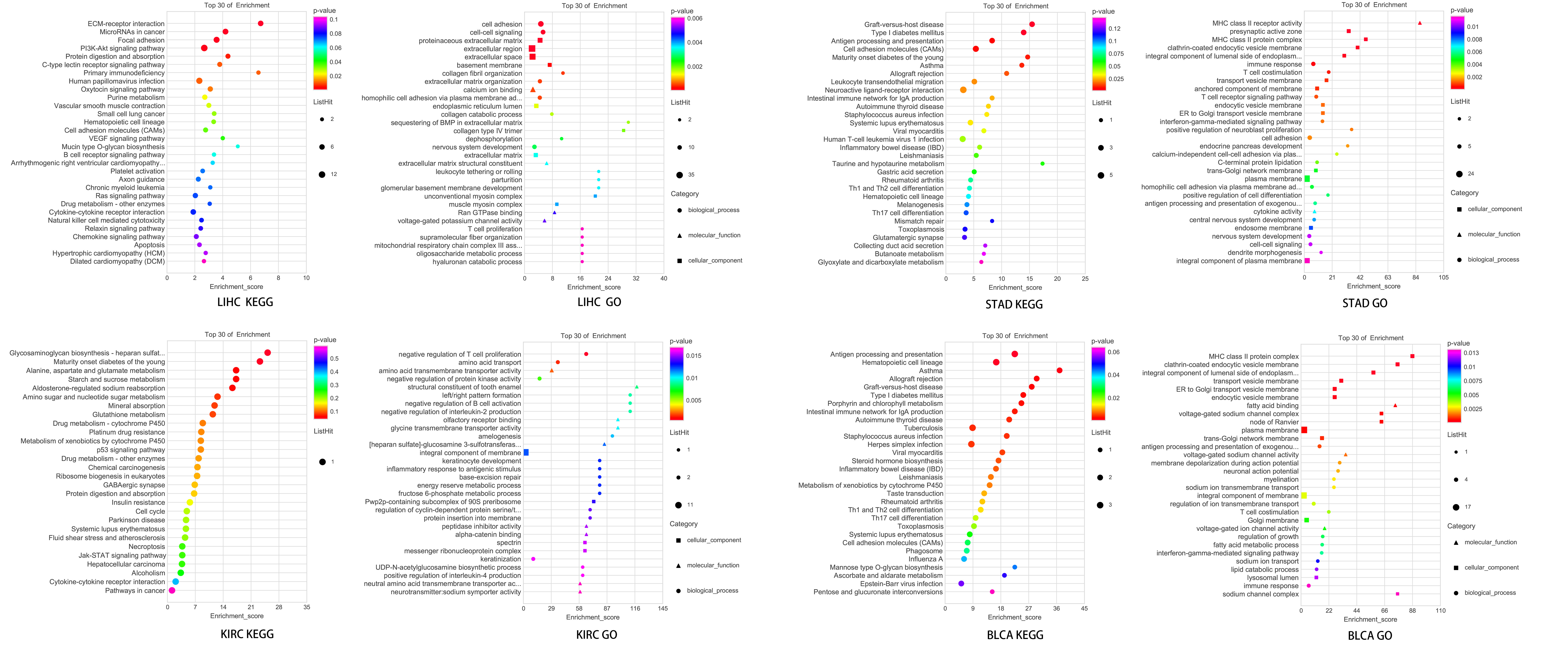

Supplement: Supplementary file 1 [file biomedicines-12-00329-s001.zip › Figure S4.jpg]
